# What anticipatory coarticulation in children tells us about speech motor control maturity

**DOI:** 10.1371/journal.pone.0231484

**Published:** 2020-04-14

**Authors:** Guillaume Barbier, Pascal Perrier, Yohan Payan, Mark K. Tiede, Silvain Gerber, Joseph S. Perkell, Lucie Ménard

**Affiliations:** 1 Grenoble INP, CNRS, GIPSA-Lab UMR 5216, Univ. Grenoble Alpes, Grenoble, France; 2 Grenoble INP, CNRS, TIMC-IMAG UMR 5525, Univ. Grenoble Alpes, Grenoble, France; 3 Haskins Laboratories, New Haven, Connecticut, United States of America; 4 Boston University, Boston, Massachusetts, United States of America; 5 Massachusetts Institute of Technology, Cambridge, Massachusetts, United States of America; 6 Department of Linguistics, Université du Québec à Montréal, Montréal, Québec, Canada; Australian Research Council Centre of Excellence in Cognition and its Disorders, AUSTRALIA

## Abstract

**Purpose:**

This study aimed to evaluate the role of motor control immaturity in the speech production characteristics of 4-year-old children, compared to adults. Specifically, two indices were examined: trial-to-trial variability, which is assumed to be linked to motor control accuracy, and anticipatory extra-syllabic vowel-to-vowel coarticulation, which is assumed to be linked to the comprehensiveness, maturity and efficiency of sensorimotor representations in the central nervous system.

**Method:**

Acoustic and articulatory (ultrasound) data were recorded for 20 children and 10 adults, all native speakers of Canadian French, during the production of isolated vowels and vowel-consonant-vowel (V_1_-C-V_2_) sequences. Trial-to-trial variability was measured in isolated vowels. Extra-syllabic anticipatory coarticulation was assessed in V_1_-C-V_2_ sequences by measuring the patterns of variability of V_1_ associated with variations in V_2_. Acoustic data were reported for all subjects and articulatory data, for a subset of 6 children and 2 adults.

**Results:**

Trial-to-trial variability was significantly larger in children. Systematic and significant anticipation of V_2_ in V_1_ was always found in adults, but was rare in children. Significant anticipation was observed in children only when V_1_ was /a/, and only along the antero-posterior dimension, with a much smaller magnitude than in adults. A closer analysis of individual speakers revealed that some children showed adult-like anticipation along this dimension, whereas the majority did not.

**Conclusion:**

The larger trial-to-trial variability and the lack of anticipatory behavior in most children—two phenomena that have been observed in several non-speech motor tasks—support the hypothesis that motor control immaturity may explain a large part of the differences observed between speech production in adults and 4-year-old children, apart from other causes that may be linked with language development.

## Introduction

Speech production in children differs from that in adults in various ways: (1) it is more variable temporally and spatially [1–11]; (2) it is slower [4,7,12,13]; and (3) the amount of anticipatory coarticulation within consonant-vowel (CV) syllables is different, although this remains controversial. Evidence for **more** coarticulation in children than in adults has been reported by some studies [9,14–17], whereas evidence for le**ss** coarticulation in children has been reported by others [11,18–22], and evidence for the **same** amount of coarticulation has been reported by some others [6,23–25]. Interestingly, a recent study reported that German speaking children and adults had similar trends in the variation of the degree of coarticulation across consonants [26].

Differences in speech production between children and adults may originate at different levels of speech production and speech perception processes [27], since (1) the units of language vary during ontogenetic development (as summarized by [28]; (2) the characterization of speech motor goals [29] and the perception of speech sounds [30–33] are different in children and adults; (3) motor control abilities become adult-like only in middle to late adolescence [34], including speech motor control abilities [3, 7, 35]; and (4) children have to deal with a vocal apparatus that is still growing, evolving non-linearly in size and shape [36–39].

In this paper, we present the results of an experiment to test the hypothesis that motor control immaturity may explain a large part of the differences in speech production between children and adults. Immaturity can be measured in a variety of ways. In the current study we rely on indices that have been used and validated for years in studies of motor control in general. Measuring these indices enables quantifying the extent to which differences between children and adult speech production originate in this immaturity. These indices are a crucial piece of our methodology and they are described and justified below.

### I. Theoretical background and working hypotheses

#### A. Indices of speech motor control immaturity

Many characteristics of children's movements, as compared to adults’ ones, appear to provide evidence of motor control immaturity. Two of them, namely the greater variability in repetitions of a single task, also called trial-to-trial variability, and the lack of effectiveness in anticipating movements, seem particularly relevant in the context of speech production [40].

*1*. *Trial-to-trial variability*. Several studies comparing the performance of children and adults in performing the same simple motor tasks have shown that the development of motor control from childhood to adulthood is associated with a significant reduction in trial-to-trial variability. For example, in their study of 54 children to investigate the role of visual feedback during the execution of a pointing task, Brown et al. (1986) [41] found that when visual feedback was fully available, the standard deviation of the positions reached at the end of the hand movement decreased monotonically and was reduced four-fold from the age of 2 years to the age of 8 years. In a similar study, Kuhtz-Buschbeck et al. (1998) [42] showed that in a grasping task, the variability of hand trajectory and grip size in a group of 6- to 7-year-old children was significantly larger than in a group of adults (see also [43]. This larger variability in children was assumed to arise from a combination of immature functions involved in motor control, that is, less efficient motor coordination [44], less efficient processing of feedback information [42], and either a larger amount of neural noise due to insufficient myelination and smaller axon diameter affecting neural transmission, or to a smaller neuron population [45].

Phonetic studies in children have revealed similar variability in speech motor control, specifically lip and jaw movements. Sharkey & Folkins (1985) [46] observed that in groups of children who were 4, 7, or 10 years old and a group of adults, during repetitions of [mæ] and [bæ] syllables, the children presented significantly more variability in lip and jaw movement amplitudes and in their temporal coordination. Interestingly, no differences were found in jaw movement amplitude in the three groups of children, but lip movement amplitude was significantly larger for the 4-year-old children than for the 7-year-old children. Smith & Goffman (1998) [3] found similar results for lip movements, characterized using their “spatiotemporal index” (STI), for repetitions of the sentence “Buy Bobby a puppy”. Four-year-old children had significantly larger STIs than 7-year-old children (30% larger) or adults (75% larger), and 7-year-old children had STIs that tended to be larger than those of adults. These two studies suggest that the reduction of articulatory variability across age is non-linear with a faster reduction from 4 to 7 years of age than from 7 onward. Interestingly, Smith & Zelaznik (2004) [7] examined the coordination of the upper lip, lower lip, and jaw for five groups of children (ranging in age from 4 to 14 years) and a group of adults in repetitions of the sentence “Buy Bobby a puppy” and “Mommy bakes pot pies” and showed that the patterns of coordination were still significantly more variable for the 14-year-old subjects than for adults (see also [35]. As with the reduction in trial-to-trial variability in arm movements in motor tasks, the reduction of trial-to-trial variability in speech kinematics during development may be explained in large part by inaccuracy in representations of motor goals or motor plans and by inaccurate processing routines, or it could also be related to inaccurate internal representations of the phonological categories, or an inaccurate or immature categorical perception of them [32,30,33,47].

*2*. *Effectiveness in anticipating movements*. In light of well-acknowledged models of serial order motor control [48] with motor systems having an excess of degrees of freedom [49,50–51] we consider that a lack of accuracy in anticipating movements is an index of motor control immaturity. This applies to speech production since it is a serial-order motor task achieved with vocal tract articulators whose effects on crucial phonetic characteristics of the speech sounds are highly redundant (see [52]). In this section, we will explain the theoretical and experimental foundations of our approach.

Since the seminal publication by Lashley (1951) [48] it is generally accepted that serial-order motor tasks require the generation of a plan in the central nervous system that specifies the whole sequence of goals and their order. Thus, the central nervous system is assumed to know all the goals and their sequencing before motor execution is launched. Lashley (1951) [48] suggested that the correct achievement of the task is obtained from the plan, due to a series of inhibitions and activations of the goals in the appropriate order with the appropriate timing. For motor systems that have an excess of degrees of freedom, i.e. control parameters that, according to the concept of motor equivalence (see for example [53]), can take different values during the execution of the same task without affecting the output, Rumelhart & Norman (1982) [49] proposed that the series of activations/inhibitions suggested by Lashley (1951) [48] could occur on separate channels in parallel, in order to take advantage of the differences in the constraints applied to the individual motor components. Building on this suggestion, Jordan (1986) [50] introduced a “parallel distributed processing” (PDP) model of serial-order motor control. According to this model [50–51], the central nervous system may use a “sensorimotor map” for motor planning, which is a representation of how each individual motor component (i.e. each vocal tract articulator in speech production) affects the realization of the successive goals of the motor task. Thanks to this sensorimotor map, before movement execution, the central nervous system can estimate for each goal, which motor component is crucial for the correct achievement of the goal, and which ones are less important. This estimation enables anticipation of movements of each of the articulators towards the achievement of upcoming goals, as long as those movements don’t produce adverse acoustic consequences. Such anticipation enables slower and smoother displacement of motor components over time, which is compatible with the reduction of effort and the preservation of the accuracy of the movements (see [54] for details about the link between speed and articulatory effort in speech production). In this theoretical framework, we consider that effectiveness in anticipating movements is a measure of motor control maturity since it reflects the capacity to take advantage of motor equivalence phenomena, in order to deal with parallel processing of serial-order motor tasks. This capacity requires that sensory motor maps have been learned with enough accuracy from a sufficiently large number of variable realizations of each of the speech motor tasks. Since children, as compared to adults, have experience with a smaller set of less varied and differentiated motor tasks, under a smaller range of conditions, we assume that in children the sensorimotor maps are less accurate than in adults, which would result in less effective and less reliable predictions of the consequences of motor commands on movements.

This hypothesis is supported by a number of experimental findings. For example, Forssberg et al. (1992) [34] investigated motor task anticipation in 10 adults and more than 90 children aged 1 to 15 years (in seven age groups) during repetitions of a task in which the subject had to grip and lift an object whose weight changed in an unpredictable manner from trial to trial. The authors observed that as of 2 years of age, all subjects tended to anticipate an object's weight and modulating their grip and lift forces as a function of the expected weight. However, while adults were very accurate and immediately corrected their grip and lift force once they realized that their anticipatory adjustments were not appropriate, children younger than 6 were very inaccurate and were unable to correct their lift forces once the lift had started. In the same vein, Bard et al. (1990) [55] measured the accuracy of hand movements towards visual goals with and without visual feedback, in groups of children aged 6, 8, and 10 years old. The authors hypothesized that while movements with visual feedback could largely rely on on-line feedback corrections, movements without feedback would have to rely on predictions based on sensorimotor maps in the central nervous system. A significant quasi-linear improvement of the accuracy in amplitude was observed from age 6 to 8 to 10 when visual feedback was not available. These two studies provide convincing examples of results that are consistent with the idea that children below 6 years of age do not have sensorimotor maps that enable them to estimate accurately the consequences of motor control commands or their interaction with the physical world.

#### B. Working hypotheses

In aiming to compare the speech motor performances of children below age 6 with that of adults, and to explain any observed differences, we chose to study 4-year-old children for two main reasons: (1) it is generally acknowledged that these children have acquired some representations of the phonemes of their language [28,56–58], and (2) from a motor control perspective, based on the studies described earlier, age four is around the onset of a period during which sensorimotor representations are beginning to play an increasing role in motor planning and motor control (see also [45,59]). Consistent with the emergence of phonemic representations at this age, we assume that 4-year-old children have moved, or are in the process of moving, from a relatively simple holistic representation of words [60], suitable for the storage of small lexicons in early development, to a more complex representation in which individual phonemes also play an important role, in later phonological development [61]. Thus, it seems reasonable to assume that, as in adults, speech production in 4-year-old (French-speaking) children is a serial-order motor task composed of a sequence of goals in which phonemes are represented, possibly with other larger units such as syllables (see [62], for an experimental support for this co-existence), and significantly influence the temporal articulatory coordination of the sequence.

In line with the theoretical rationales developed above, we consider that anticipatory coarticulation, a crucial characteristic of adult speech associated with speech motor planning [63], results from the use of complex, advanced components of planning: (1) refined speech sensorimotor maps that enable speakers to take advantage, in sequence planning, of freedom associated with possibilities for motor equivalence (see [52]), and (2) efficient parallel processing of the control of each articulatory component, which integrates the motor plan (the goals and their serial-order) and the different constraints acting on the articulators. Importantly, in this context we hypothesize that 4-year-old children do not have accurate speech sensorimotor maps covering the whole motor command space, because of their incomplete experience of the sensory consequences of motor control; consequently, children are less able to deal with anticipatory coarticulation, as adults do.

These assumptions are not based on straightforward inferences from previous research, since investigations of coarticulation patterns in speech produced by children compared to adults have reported conflicting results (see for example [14] *versus* [11] *versus* [25]). However, we believe that these conflicting results may arise from the fact that most of these studies focused on coarticulation within the syllable. Indeed, syllables produced by 4-year-old children, which are the main units of babbling and of bi-syllabic first words, may still be represented in a holistic manner, for example in the form of a motor program or a gestural score specifically dedicated to the production of a syllable, or they may be represented as a sequence of phonemes, since at this age children are in the middle of a cognitive process transforming their representations from a holistic to one that includes a segmental component (see [28], for a summary of related studies). We hypothesize that in a group of 4-year-old children, coarticulation patterns within a syllable may vary significantly across subjects, since some of the children may be able to control syllables as serial-order motor tasks, whereas others would still rely on a holistic specification, with the possibility that both representations coexist in a subject, with different weights, as suggested by Caudrelier et al. (2019) [58].

For these reasons, we decided to focus the study of anticipatory coarticulation on V_1_CV_2_ sequences, and to analyze how the production of V_2_ influences the production of V_1_ across the boundaries of the CV syllable. In our view considering coarticulation over a sequence of phonemes that are located on both sides of the syllable boundary increases the likelihood that mechanisms underlying serial-order motor control are at play, rather than holistic motor programs remaining from the first stage of language development.

#### C. Summary

To summarize, the present study aimed at evaluating whether children show evidence of less mature speech motor control than adults, as indexed by greater trial-to-trial variability in repetitions of a simple motor task, namely the production of isolated vowels, and by less anticipation of vowel V_2_ in vowel V_1_ within V_1_CV_2_ isolated sequences. We hypothesized that compared to adults, 4-year-old children would show significantly larger trial-to-trial variability because of their immature motor control accuracy, and they would also show a significantly smaller influence of V_2_ on V_1_ because of their immature sensorimotor representations in the central nervous system and possibly because of their immature capacity to deal with parallel processing of serial-order motor tasks.

## II. Materials and methods

### A. Participants

Twenty 4-year-old French Canadian children (aged 4 years 0 months to 4 years 11 months; 9 boys) and 10 French Canadian adults (aged 19 to 30 years old; 4 males) were recruited in Montréal. All participants were native speakers of Canadian French and did not use any other language. All children lived in monolingual French families and were educated in French only. Most children had parents with university degrees. Participants reported no history of speech, language or hearing problems. All participants had normal hearing, as shown by a bilateral pure tone screening test at 20dB at 250Hz, 500Hz, 1000Hz, 2000Hz and 4000Hz before the experiment. All adult participants and the parents of the child participants were informed about the procedures before the experiment and gave their consent and the study was approved by the ethical committee of the Université du Québec à Montréal (UQÀM). Each child received a little gift for their participation. This paper presents the acoustic results from all participants and the articulatory results from a subset of six children and two adults.

### B. Data acquisition

**Fig 1:**
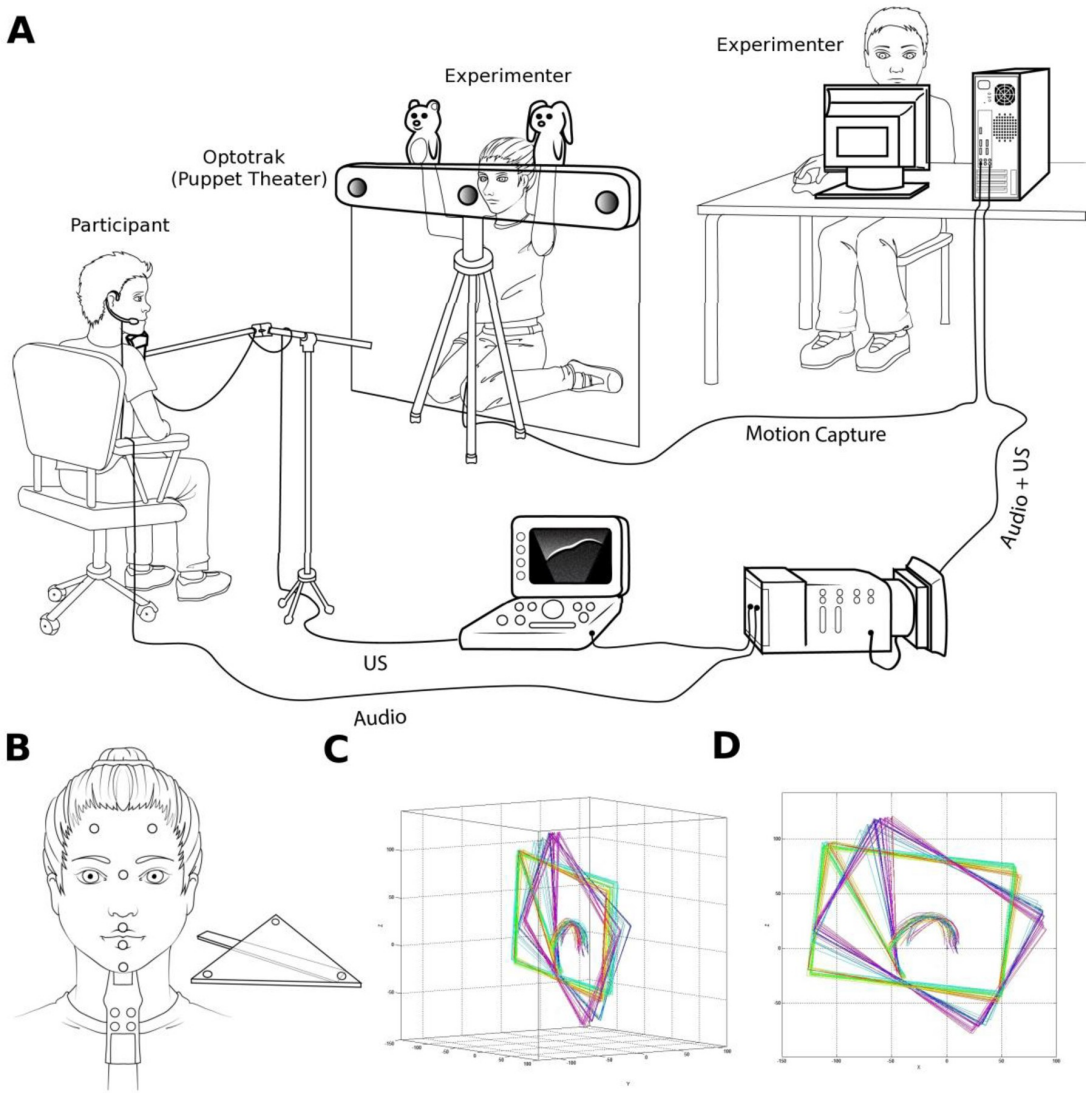
A. Experimental setup (where US = UltraSound). A participant is seated in front of the Optotrak. To keep the subject from seeing the activities of the operator who was presenting the dolls, the operator was hidden behind a sheet suspended from the Optotrack sensor bar. Synchronized ultrasound and acoustic data are recorded, as well as Optotrak motion capture data, in order to align extracted tongue contours with palatal hard structures. B. Placement of the Optotrak IREDs on the participant's head and ultrasound probe. The device used to measure the occlusal plane is also shown. (Illustrations by Sabine Burfin.) C. Ultrasound tongue contours corrected for head movements. D. The same contours projected onto the midsagittal plane. Note that between some data (green-yellow) and others (blue-purple), the child participant moved, but data were realigned within a single articulatory space, relative to the child’s hard palate.

Ultrasound is a benign noninvasive imaging technique that is suitable for use with very young children [64]. For this study midsagittal images of lingual articulation were collected with ultrasound using a probe mounted on a flexible boom microphone stand to maintain contact with submandibular skin. This approach is appropriate for developmental studies, in that it preserves some freedom of mandible movement for the participants. To obtain reliable measurements of tongue movements in relation to the palatal hard structure we simultaneously recorded sensors attached to the head and probe to track their frame-by-frame location. We also recorded lip and chin position. A schematic representation of the experimental setup is presented in Fig 1.

Synchronous recordings of tongue movements in the midsagittal plane (at NTSC 29.97 Hz) and of the speech signal (at 44.1kHz) were made using a Sonosite 180Plus ultrasound device and a directional microphone. An Optotrak system (NDI Certus) was used to record the audio signal and the positions of infrared emitting diodes (iREDs) at 100 Hz. Three iREDs were positioned on the participant's forehead and four iREDs were positioned on the ultrasound probe. At the beginning of the experiment, three iREDS mounted on a plastic triangle were used to record the orientation of the occlusal plane while the participant held it firmly between their teeth during a reference trial. A second reference trial established the jaw-clenched position of the ultrasound probe relative to the head. To record lip and jaw movements, two iREDS were glued at the midline on the vermilion borders of the upper and lower lips and one on the chin. The analysis of these lip and jaw data are not included in this study. A separate calibration session was used to establish the correspondence between the Ultrasound and Optotrak coordinate systems using fiduciary points visible on the probe surface mapped to the location of a corresponding IRED.

### C. Task

Data were collected on-site at daycare centers in Montréal and at the Laboratoire de Phonétique, UQÀM. Participants were seated in front of the Optotrak, which was disguised as a puppet theater, and the ultrasound probe was held under their chins by a microphone stand (see Fig 1). One experimenter checked that participant's heads were essentially immobile with reference to the ultrasound probe, and that most of the tongue was visible in the ultrasound image; another experimenter controlled the recording (Optotrak and ultrasound) and checked that all the iREDs were visible during the trials.

The corpus consisted of two speech tasks. First, between 8 and 10 repetitions of isolated vowels /i e ε a u/ were elicited. Those vowels were used to measure trial-to-trial variability in the F1-F2 plane without any influence of a phonetic environment. Second, between 8 and 10 repetitions of V_1_-C-V_2_ sequences were elicited with C being one of /b d g/, V_1_ one of /ε a/, and V_2_ one of /i a/. The vowels, /ε/ and /a/ were chosen for V_1_ since each can be produced with a noticeable amount of articulatory variability of the tongue without seriously affecting their perception. The extreme vowels /i/ (high, front) and /a/ (low, central) were chosen for V_2_ selected since their anticipation has been shown in French adults to significantly affect the articulation of preceding sounds [65]. Thus, V_1_-C-V_2_ sequences were designed to measure the effects of the anticipation of V_2_ in the realization of V_1_ in a context that maximizes the potential to observe such anticipation.

The target words corresponded to puppet names, as illustrated in Fig 1. The puppets were manipulated by an experimenter who was hiding behind a screen (i.e., a “theater”). The participant was instructed to say the puppet’s name each time the experimenter would show it. The target words were presented as a pair, with two different puppets each time. Before data recording, we made sure the participant could assign the right name to each puppet. This familiarization time was very short, and required no more than two trials (not recorded). Participants did not receive any kind of feedback, apart from when they assigned the wrong name to the puppet. All participants produced a minimum of 8 repetitions and a maximum of 10 repetitions of each target sequence. Only a few trials were lost (less than 10) among the total number of 3000 tokens.

The tasks were presented as puppet games, with a third experimenter serving as puppet master. The puppets' names were the isolated vowels ([u], [a]…) or V_1_-C-V_2_ sequences ([abi], [aba], [iba]….) described above. To facilitate memory retention by participants, puppets were presented in different pairs (and pairs were randomized across subjects). The order of appearance was randomized. The task was to pronounce the name of the puppet when it appeared. Thus, participants had to plan and execute a speech movement or a sequence of speech movements.

### D. Data post-processing and statistical analyses

#### 1. Acoustic data

The acoustic signal was downsampled to 16 kHz in order to achieve more accurate formant detection. This signal was first segmented (labeled) manually with *Praat* [66]. For both isolated vowels and vowels in the target sequences, vowel onset was defined as the first descending zero-crossing of the acoustic signal after the clear emergence of F2 on the synchronous wide band spectrogram, and vowel offset was defined as the first descending zero-crossing after the disappearance of F2. Automatic formant detection at vowel midpoint was carried out with a linear predictive coding (LPC) method (downsampling to 12kHz; Hanning window of 20ms; LPC order 14; with pre-emphasis) using an in-house MATLAB script. Because formant tracking is difficult in children's speech, with the potential risk for detection errors, we combined the measure of the frequencies of the maxima in the envelope of the frequency response of the LPC filter with the measure of the frequencies of the poles (computed from the angle of the pole in the upper half of the z-plane) of the LPC filter. For each vowel, a range of acceptable formant values was used to guide the selection of the correct formants among all possible candidates and remove outliers. The acoustic signal was also used to provide a measure of the duration of the segmented V_1_-C-V_2_ sequences, which we consider to be a reliable inverse indicator of the speed of average articulatory movements.

Prior to the statistical analysis, F1 values and F2 values were z-scored (one for each spectral parameter) for each speaker separately, in order to eliminate interspeaker variability associated with intrinsic morphological differences in the vocal tract. This transformation (acting like a vowel-space normalization across speakers and across ages) enables the grouping of children’s z-scored formant values and of adult’s z-scored formant values, and a comparison of adults and children on this basis. To ensure that children and adults produced distinct vowel categories and thus achieved the task, for each participant, a linear discriminant analysis was conducted with vowels as the grouping factor and z-scored F1 and F2 values as the independent variable list. Within-speaker percent correct classifications scores ranged from 93.2% to 100% and did not vary significantly as a function of speaker group.

The trial-to-trial variability in the production of the isolated /i e ε a u/ vowels was analyzed with two linear mixed effects models in which the variable to be explained was either the standard deviation in z-scored F1 values or the standard deviation in z-scored F2 values, computed for each subject separately across the repetitions a same vowel. The fixed effects were the speaker group (children and adults) and the vowel (/i/, /e/, /ε/, /a/ and /u/), and the intercepts and slopes by participants were considered as a random effect. These analyses were performed using the *lme4* [67] package implemented in R [68]. Visual inspection of residual plots was used to confirm the absence of any obvious deviation from homoscedasticity or normality. In the absence of deviation, the statistical analyses were considered to be valid and p-values were obtained by likelihood ratio tests of the full model with the effect in question versus the model without the effect in question.

Regarding the second task (production of V_1_CV_2_ sequences), used to measure anticipatory coarticulation, for each of the two vowels V_1_ (/a/ and /ɛ/), the z-scored formant values F1 and F2 were extracted at vowel midpoint. The effect of V_2_ (/i/ or /a/) on V_1_'s formant values was assessed through linear mixed effects modeling using speaker group (children and adults), V_1_ (/a/ and /ε/) and V_2_ (/i/ and /a/) as fixed factors. Intercepts and slopes by participants were entered as random effects. Visual inspection of residual plots was used to confirm the absence of any obvious deviation from homoscedasticity or normality. In the absence of deviation, the statistical analyses were considered to be valid and p-values were obtained by likelihood ratio tests of the full model with the effect in question compared with the model without the effect in question. For significant interactions, multiple comparisons were conducted using the *glht* function of the *multcomp* package [69]. Random effects were further explored by comparing models built with random intercepts only to models built with random intercepts and slopes. This allowed us to examine participant-specific behavior. When significant differences were found, each participant’s average values were considered and interpreted (*ranef* function).

Last, the effects of V1 and V2, and of speaker group on the sequence duration were analyzed through a linear mixed effects model in which speaker group, and V1 (/a/ and /ε/) and V2 (/i/ and /a/) were the fixed effects. The intercepts and slopes by participants were included as a random effect.

#### 2. Ultrasound images

Tongue contours were fit to ultrasound images from times corresponding to vowel V_1_ midpoints using an interactive spline fitting procedure (*GetContours*; [70]). Each extracted contour was made up of 100 equally-spaced pixels in the 2-D coordinates of the image space. Using the calibration session described above, these were first converted to 3D Optotrak coordinates, then aligned to palatal hard structure by adjusting for head and probe displacement relative to the reference trials (HOCUS; [71]).

Trial-to-trial variability of isolated vowels in the articulatory domain was measured via the "nearest neighbor distance" as described by Zharkova et al. (2011) [9]. The mean distance between two tongue contours corresponding to repetitions of a given vowel was computed on the basis of the average point-by-point Euclidean distance between the 100 points of each of the tongue contours. Because this measurement is highly dependent on the positions of the beginning and end of the tongue contours visible on the ultrasound images, it was only used to quantify within-category variability (for which the beginning and end of tongue contours are comparable). Linear mixed effects analysis of the relationship between articulatory distance, the variable to be explained, and speaker group and vowel as fixed effects were performed using *lme4*. Intercepts and slopes by participant were entered as random effects. Visual inspection of residual plots was used to confirm the absence of any obvious deviation from homoscedasticity or normality. In the absence of deviation, the statistical analyses were considered to be valid and p-values were obtained by likelihood ratio tests of the full model with the effect in question compared to the model without the effect in question.

Concerning the V_1_CV_2_ sequences, two metrics were used to compare tongue position and shape for each V_1_ vowel (/a/ and /ε/) across V_2_ contexts. First, based on previous work focusing on the development of anticipatory coarticulation ([26], for instance), for each tongue contour, the (x,y) coordinates of the highest point of the contour were extracted. These values were z-scored in order to cancel the influence of vocal tract size differences between adults and children. Although this method has been used previously and follows standard phonetic descriptions of vowel production, it substantially reduces the information provided by ultrasound images. Thus, smoothing spline ANOVAs [72] were performed [73]. This method provides for each speaker group a comparison of sets of tongue contours measured for each vowel V_1_ in the same V_2_ context by constructing confidence intervals around the average contours.

**Fig 2:**
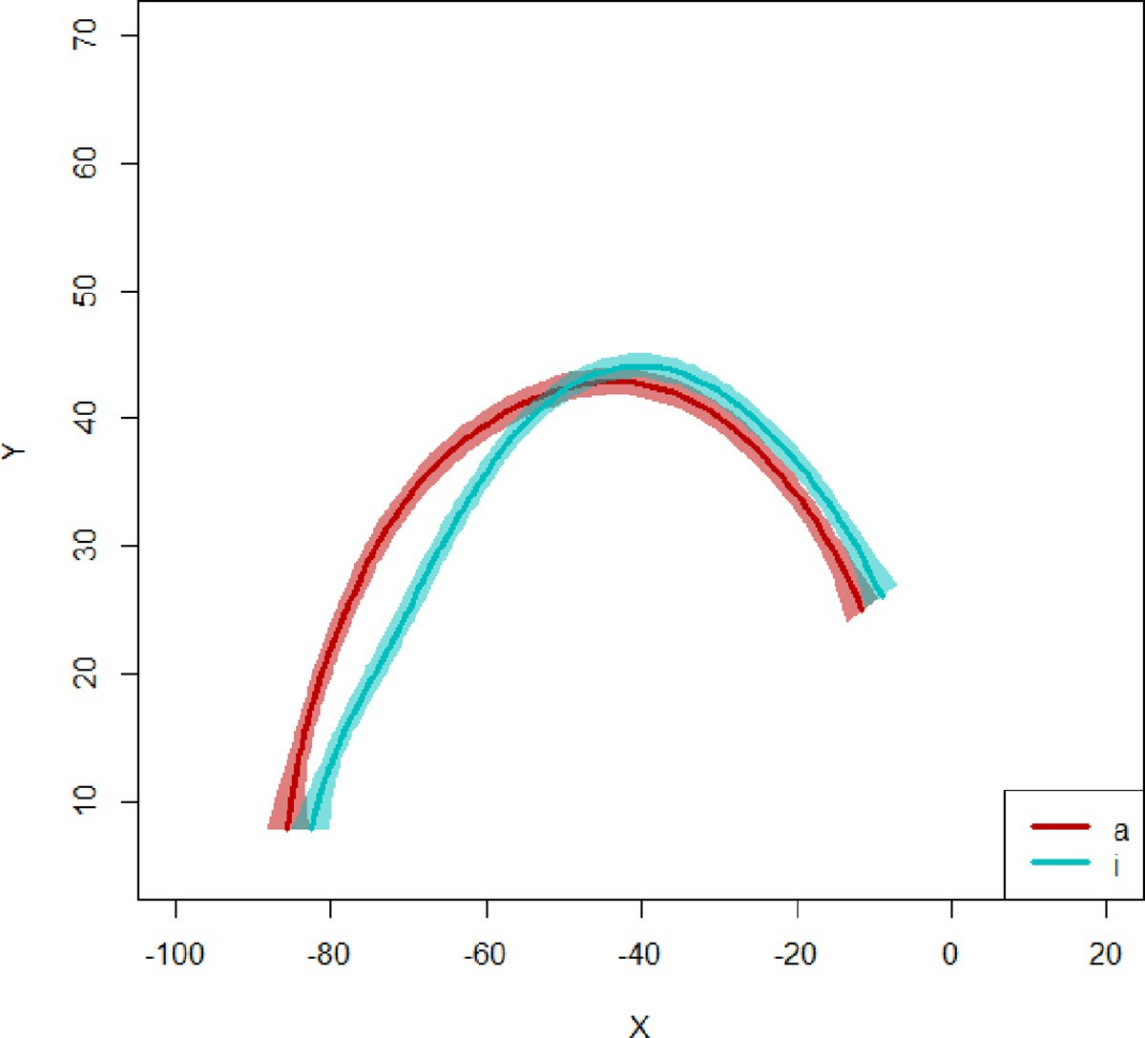
**Illustration of average splines corresponding to midsagittal tongue contours with 95% confidence intervals, for a child participant, for /ε/ in /εbi/ (blue) and in /εba/ (red).** X and Y are in mm.

In the current study, we used 95% confidence intervals to threshold the variability of the data. Two sets of contours were compared for each V_1_ (/a/ or /ε/) and consonantal context: one corresponding to tokens for which V_2_ is /i/, and one corresponding to tokens for which V_2_ is /a/. Fig 2 shows the splines for a representative child participant. To evaluate the extent that the two sets of contours differ according to V_2_, the proportion of points along the contours for which both confidence intervals overlap was calculated. The figure shows the average splines as well as the variability across repetitions (95% confidence intervals).

As was the case for the acoustic parameters F1 and F2, the effect of V_2_ on the tongue position and shape parameters in V_1_ discussed above was assessed through linear mixed effects models using the *lme4* package.

## III. Results

### A. Trial-to-trial variability in isolated vowels

**Fig 3:**
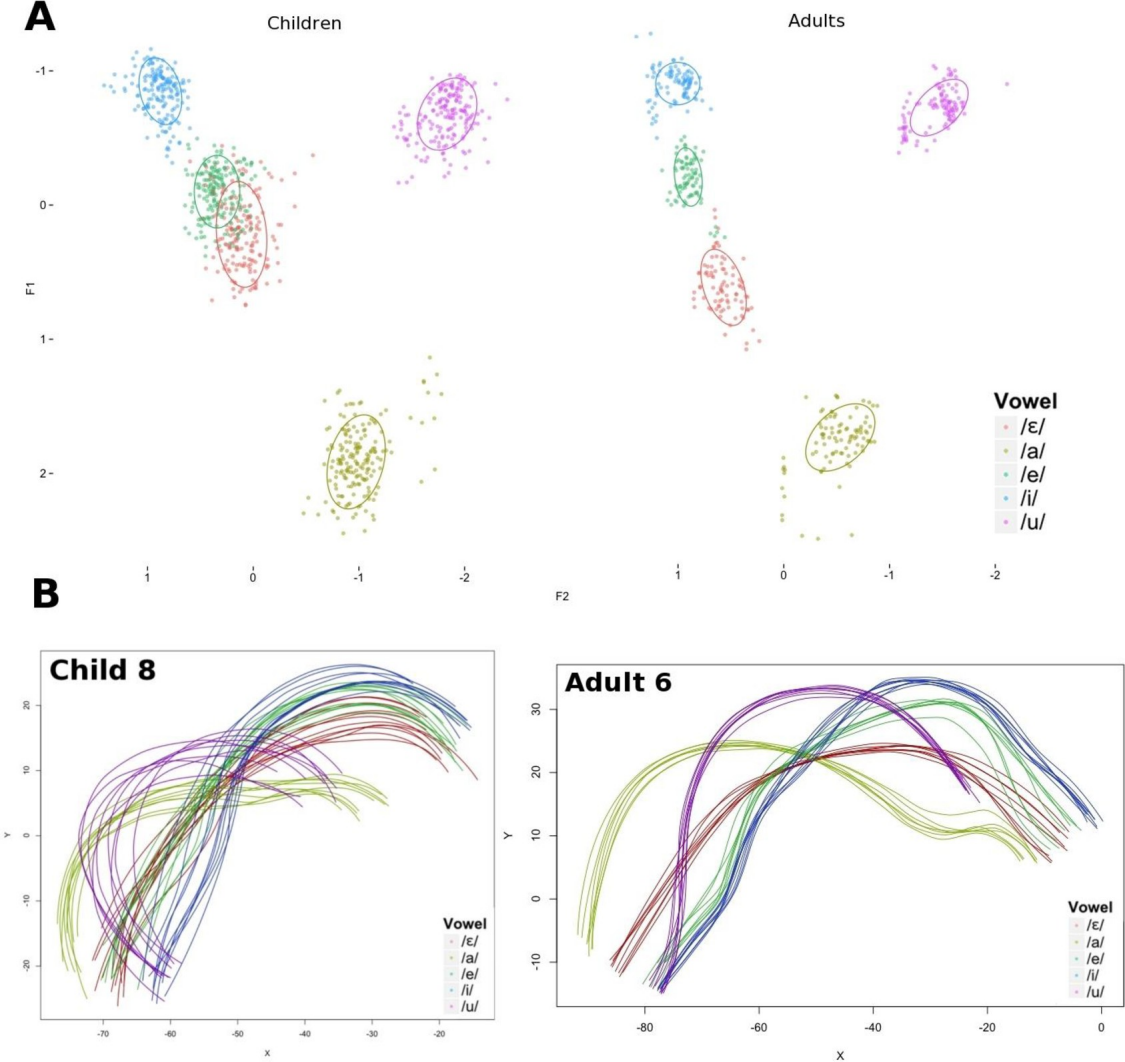
Illustration of the trial-to-trial variability in vowel production in the acoustic and articulatory domains, and the main differences between the group of adults and the group of children. Top panels: Variability in the z-scored (F1, F2) planes for the group of children (left) and the group of adults (right). Bottom panels: Examples of articulatory variability in the mid-sagittal plane for a child (left) and an adult (right) participant; X and Y are in mm.

Fig 3 illustrates the main trends observed in the acoustic and articulatory domains for the trial-to-trial variability and the differences between the group of children and the group of adults. Children clearly presented more variability than adults in both domains. A more specific analysis of these results is presented in the rest of this section.

**Fig 4:**
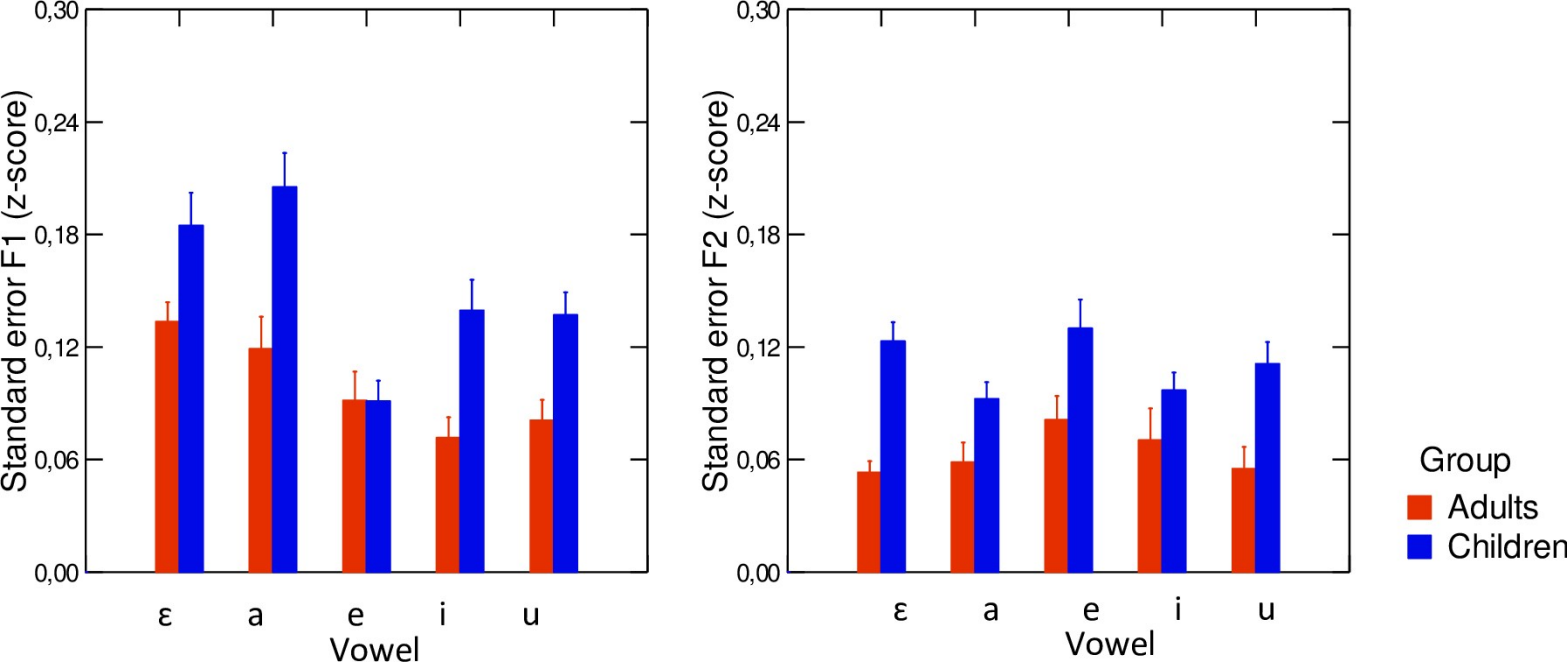
**Average values of standard error of z-scored formants F1 and F2 for each vowel category, across speaker groups (left-hand panel: F1, right-hand panel: F2).** Red columns correspond to adult participants and blue columns correspond to child participants. Error bars are standard errors of the mean.

The average values of standard deviations measured on z-scored F1 and F2 values across all repetitions for each of the /i e ε a u/ vowel categories averaged within speaker groups in the acoustic domain are displayed in Fig 4. Results of the linear mixed effects models conducted separately on z-scored F1 and F2 revealed significant effects of speaker group both as a main effect and in interactions with vowel category. The significant effect of speaker group on F1 variability (χ^2^(5) = 22.56; p<0.001) showed that children had larger standard deviation values than adults. In addition, the analysis revealed a main effect of vowel category on F1 variability (χ^2^(8) = 49.55; p<0.001), in which /i u e/ had significantly lower variability than /a ɛ/ (p<0.05). As for the variability in F2, a significant main effect of speaker group was also found (χ^2^(5) = 21.52; p<0.001); children had larger standard deviation values than adults, for all vowels under study. Thus, trial-to-trial variability in the acoustic domain varied significantly across groups.

**Fig 5:**
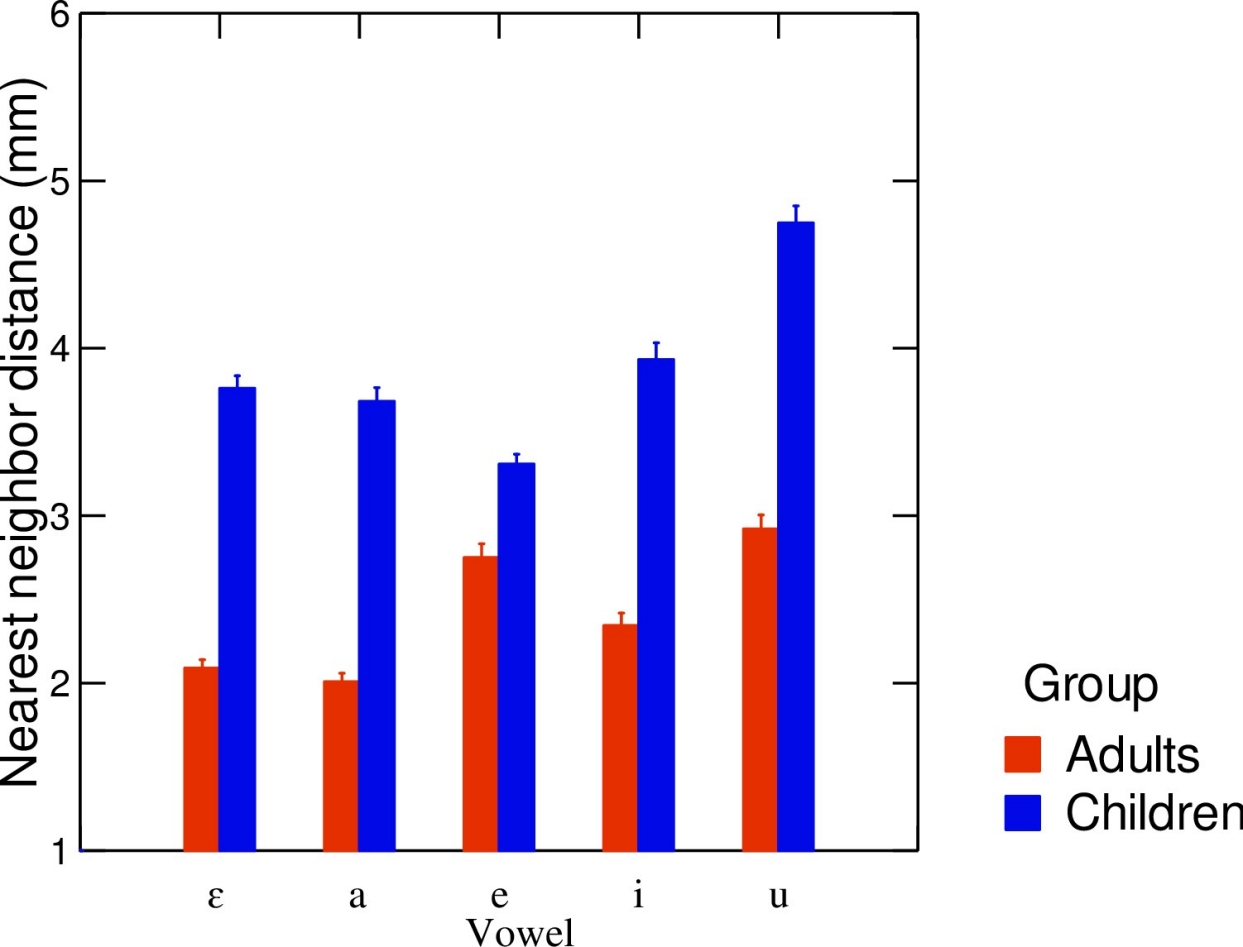
Average nearest neighbor distance for each vowel category, across age groups. Error bars are standard errors of the mean.

In the articulatory domain, "nearest neighbor distance" values averaged across speaker groups and vowel categories are shown in Fig 5. The average trial-to-trial variability nearest neighbor distance in adults was 2.4 mm, ranging from 1.7 to 3.0 mm. The average trial-to-trial variability in nearest neighbor distance in children was 3.9 mm, ranging from 3.3 to 4.4 mm. A linear mixed-effect model conducted on the average standard deviation revealed that children had significantly higher nearest neighbor distance values (i.e. larger trial-by-trial articulatory variability) than adults (χ^2^(5) = 18.03; p<0.001). A significant effect of the interaction between vowel category and speaker group was found (χ^2^(4) = 16.89; p<0.001), where the group difference was smaller for /e/, compared to the other four vowels. Combined with the acoustic results, the higher variability observed for children across repetitions in the articulatory domain suggests that the stability of the control was greater in adults than in children. This will be further discussed below.

### B. Anticipation of V_2_ in V_1_ within V_1_CV_2_ sequences

#### 1. Duration of V_1_CV_2_ sequences

**Fig 6:**
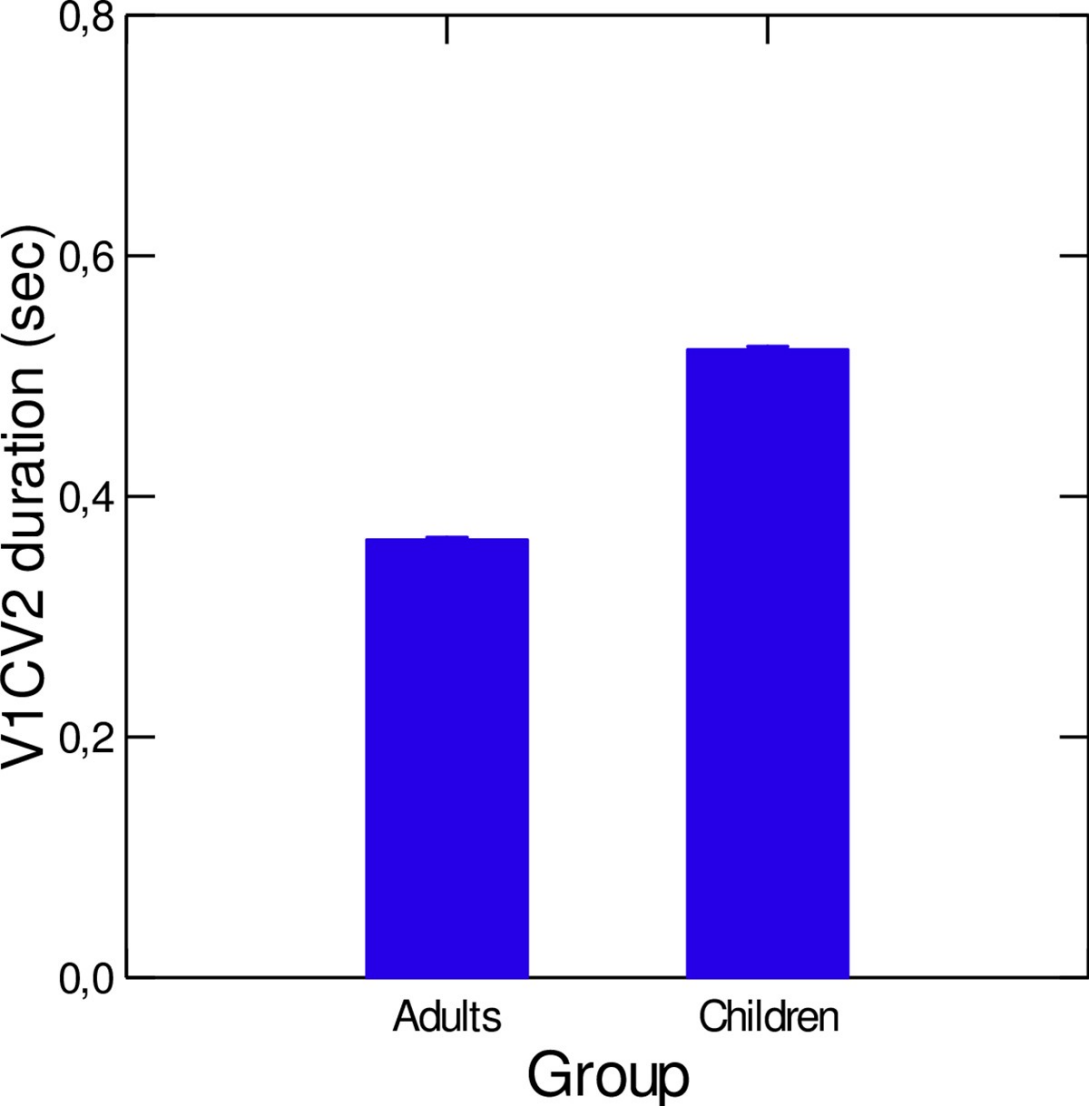
Average duration of V_1_CV_2_ sequences, for both speaker groups. Error bars are standard errors of the mean.

The average duration of V_1_CV_2_ sequences for both speaker groups is depicted in Fig 6. Not surprisingly (see Introduction), the average duration of the sequences was significantly larger in children (0.536 sec) than in adults (0.362 sec) (χ^2^(1) = 14.03; p<0.001).

#### 2. Acoustic domain

**Fig 7:**
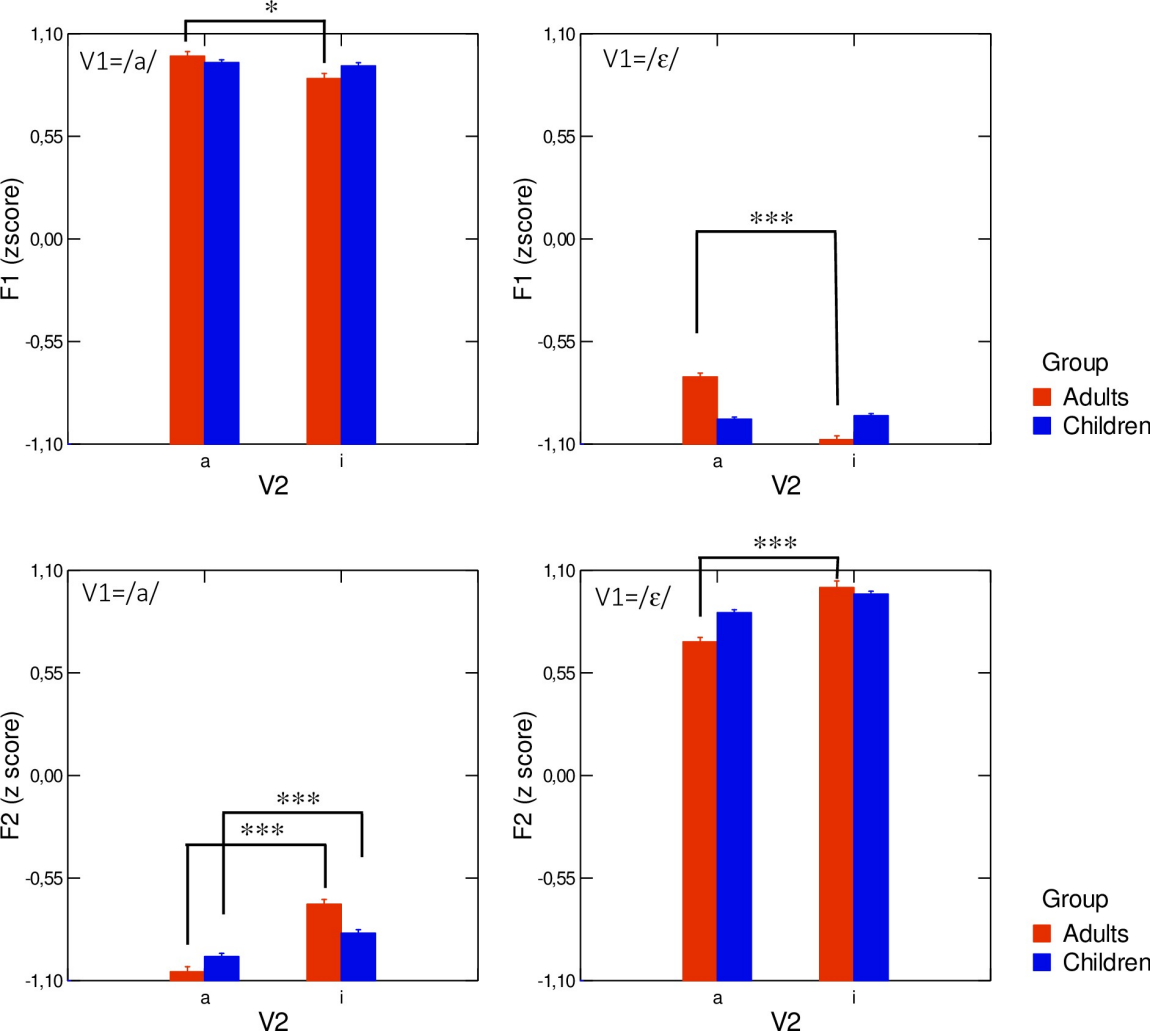
Average values of z-scored F1 and F2, for both speaker groups and V_2_ contexts, in V_1_CV_2_ sequences. Error bars are standard errors of the mean.

Average values of z-scored F1 and F2 measured at V_1_ midpoint are presented for both speaker groups and V_2_ contexts in Fig 7. In this figure, the effect of V_2_ on V_1_ can be measured through the difference in V_1_ formant values depending on the upcoming vowel. If the formant values differ from one context to another, and if this difference occurs in the direction of the upcoming vowel V_2_, we conclude that there is an anticipation of V_2_ in V_1_.

Fig 7 and Table 1 show that there were clear anticipatory effects for adults: for both V_1_ vowels (/a/ and /ε/), F1 was lower and F2 was higher when V_2_ = /i/ than when V_2_ = /a/. For children, some trends were observed, but they were weaker and not always compatible with anticipation. The statistical analysis based on linear mixed-effects models carried out separately on F1 and F2 reinforced these preliminary qualitative observations. A significant interaction existed both for F1 and F2 between the fixed effects speaker group, V_1_ and V_2_ (F1: χ^2^(4) = 119.09; p<0.001—F2: χ^2^(4) = 88.25; p<0.001). Results of multiple comparisons of z-scored formant values performed on V_2_ levels, within speaker groups and V_1_ levels are presented in Table 1. For adults, significant anticipation was found in V_1:_ F1 in V_1_ was significantly lower and F2 was significantly higher when V_2_ = /i/ than when V_2_ = /a/ (see top 4 rows in Table 1). For children, no anticipation was found in V_1_ when considering F1. However, when considering F2, the results depended on V_1_: if V_1_ = /a/ a significant anticipation was observed (7^th^ row in Table 1) whereas for V_1_ = / ε /, there was a non-significant trend for anticipation.

**Table 1:** Results of multiple comparisons of z-scored formant values performed on V_2_ levels, within groups and V_1_.

Comparison	Estimate	Standard error	z value	Significance
**Adults F1**_**V1**_				
/aCi/ vs. /aCa/	-0.122	0.040	-3.027	*
/εCi/ vs. /εCa/	-0.340	0.045	-7.625	***
**Adults F2**_**V1**_				
/aCi/ vs. /aCa/	0.370	0.060	6.169	***
/εCi/ vs. /εCa/	0.300	0.059	5.054	***
**Children F1**_**V1**_				
/aCi/ vs. /aCa/	-0.018	0.019	-0.969	ns
/εCi/ vs. /εCa/	0.017	0.019	0.902	ns
**Children F2**_**V1**_				
/aCi/ vs. /aCa/	0.128	0.029	4.428	***
/εCi/ vs. /εCa/	0.104	0.042	2.498	ns

where ns = nonsignificant

* = p<0.05

** = p<0.01, and

*** = p<0.001

Therefore, while anticipation was significant in adults both along the open/close (F1) and the antero-posterior (F2) directions, children did not show anticipation except for when V_1_ = /a/ and formant F2 was considered. Interestingly, in this case changes in z-scored F2 associated with anticipation were clearly smaller in children (0.128) than in adults (0.370). Based on these findings, it was important to clarify whether the smaller magnitude of anticipation of V_2_ along the F2 dimension observed in children was because all children anticipated V_2_ but to a lesser extent than adults, or because some children anticipated V_2_ like adults while others did not. To investigate this, we analyzed the contribution of individual adults and children to the group data. The random effect (speaker group) was further explored by considering for each group (adults or children) the interactions between the subjects (random-effect) and V_1_ and V_2_. A comparison of the models for F2 showed that these interactions were significant for both adults and children.

**Fig 8:**
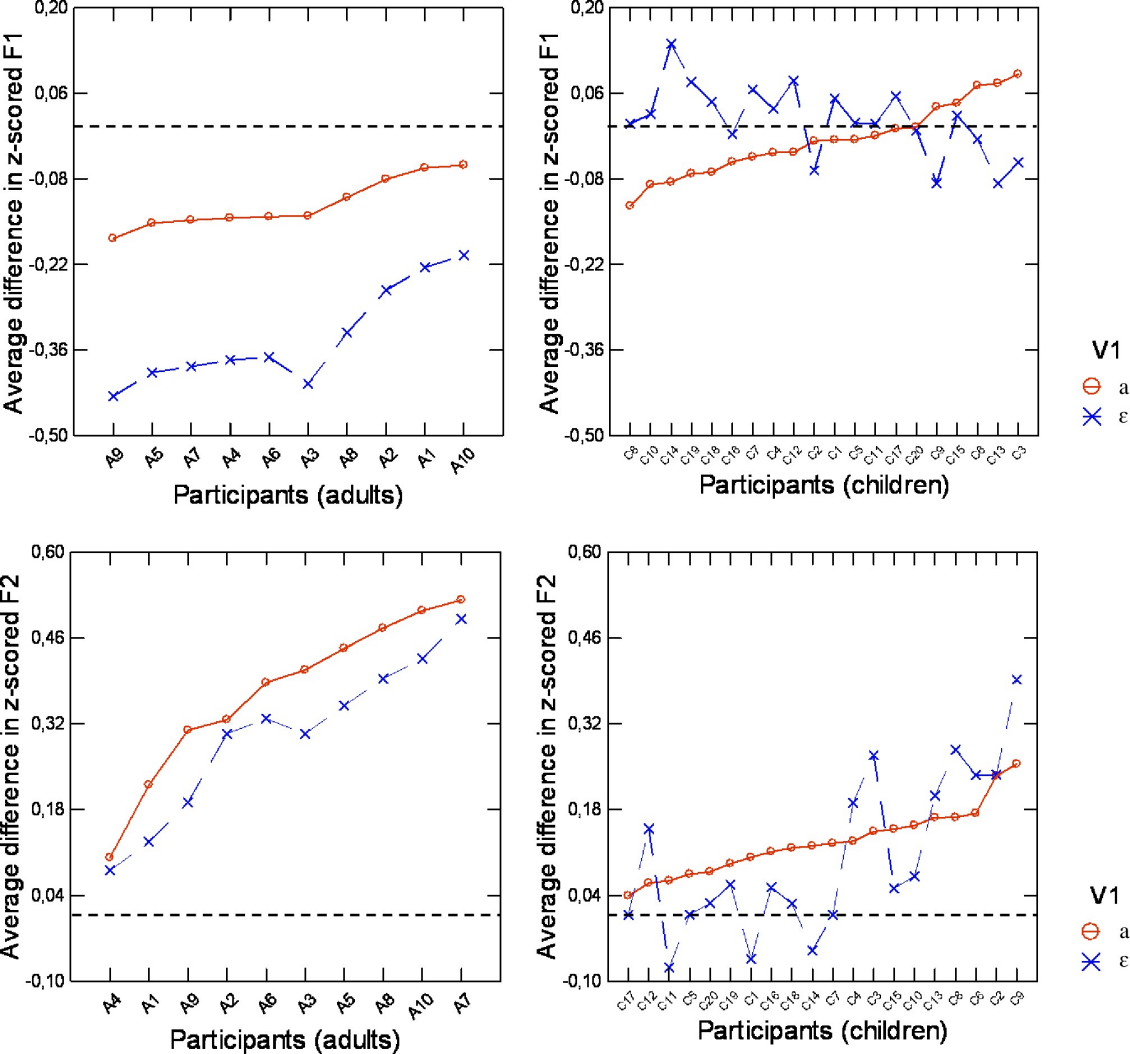
Average difference in z-scored F1 and z-scored F2 in V_1_ between V_1_C/i/ and V_1_C/a/, per participant. The solid line corresponds to /a/CV_2_ sequences and the dashed line corresponds to /ɛ/CV_2_ sequences. In each panel the horizontal dotted line indicates zero difference. In the upper panels anticipation corresponds to negative differences. In the lower panels anticipation corresponds to positive differences.

A graphic representation of average anticipation in z-scored F1 and z-scored F2 per participant is presented for each V_1_ vowel in Fig 8, for the group of adults (left-hand panel) and the child- participant group (right-hand panel). In this figure, each data point corresponds to the difference in z-scored F2 or F1 values measured in V_1_ between V_1_C/i/ sequences and V_1_C/a/ sequences. As mentioned earlier, a negative value for the differences in F1 or a positive value for the difference in F2 corresponds to an anticipation of V_2_ in V_1_. Observation of these plots lets us draw a first very important conclusion: all the adults anticipated V2 in both V_1_ vowels, since all the measured differences were clearly negative for F1 and positive for F2. On the contrary, for children patterns opposite of anticipatory behavior were often observed: this was the case of children C1, C4, C5, C7, C8, C10, C11, C12, C14, C15, C17, C18, C19 and C20 who produced positive differences in F1 for V_1_ = /ɛ/, and for children C1, C11, and C14, for which negative differences were observed in F2 for V_1_ = /ɛ/. Non-negligible differences were observed across adult speakers, since three of them produced clearly less variability in F2 than the rest of the group, but for the large majority the magnitude of the variations in F2 associated with variations in V2 was above the range of values displayed by the children (right-hand panel), for which much more between-speaker variability was observed. Four children (C8, C6, C2, and C9) exhibited large differences in F2 for both V_1_ vowels, which were in the same range as the differences observed for the adult participants. This corresponds to an adult-like anticipatory behavior. Among the other children, some did not show any significant anticipation in F2 of V_2_ (especially when V_1_ = /ɛ/ with eleven subjects, C17, C11, C5, C20, C19, C1, C16, C18, C14, C7, and C15, producing a difference smaller than 0.05), whereas others behaved like the adults who produced the smallest amount of anticipation. Thus, even though at the group level, children displayed reduced anticipatory behavior in the F2 dimension, at the individual level, children varied considerably in the extent to which they anticipated V_2_, with four children producing patterns similar to those observed in most adult participants.

Articulatory correlates of these different patterns of anticipatory behavior in children and adults were then investigated by analyzing variability in tongue positions according to two different methods: the measure of position of the highest point of the tongue, and the characterization of the whole tongue contour. Recall that the processed articulatory data come from a reduced number of subjects, compared to the acoustic data, since it is from only 2 adults and 6 children.

#### 3. Highest point of the tongue

**Fig 9:**
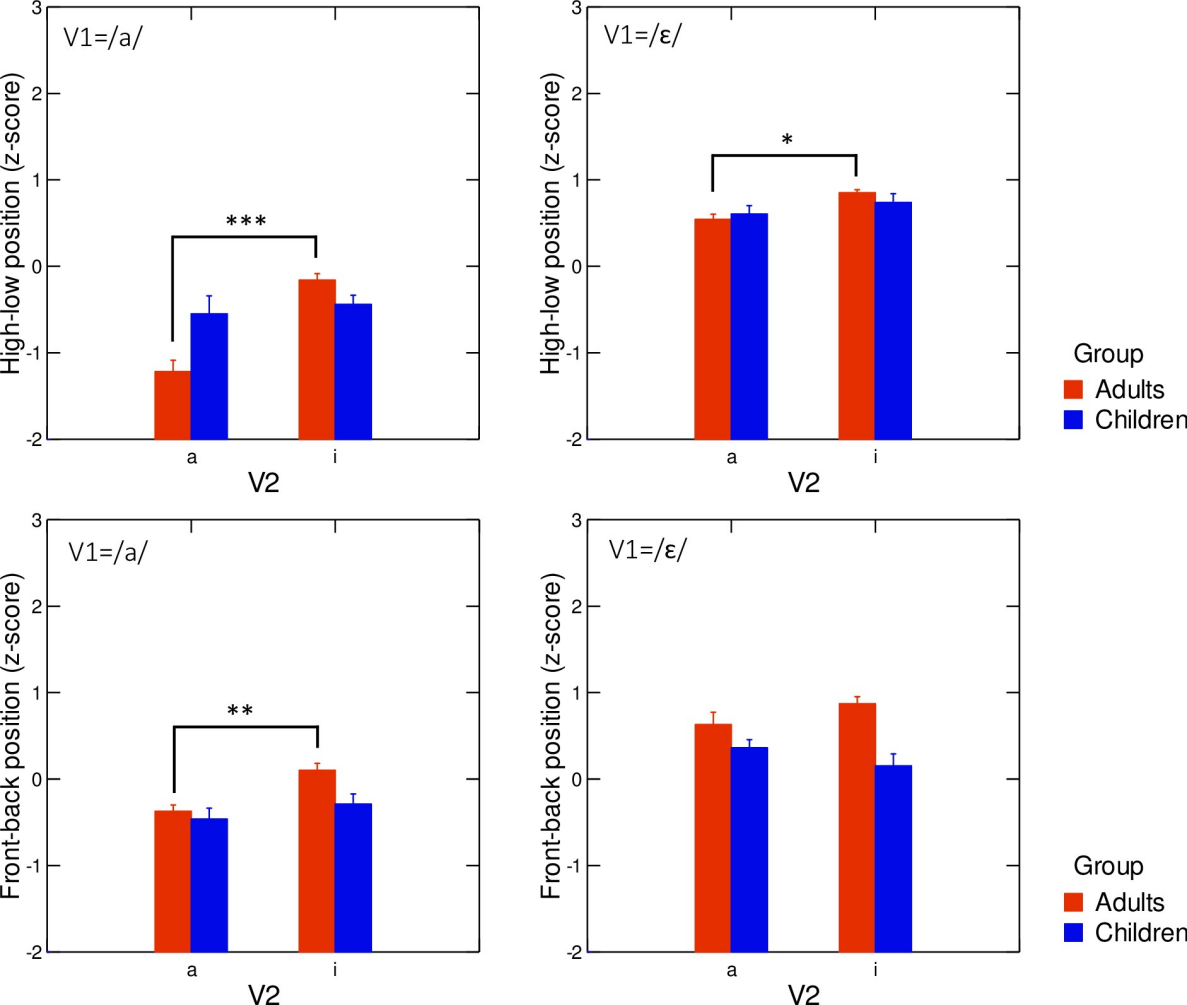
**Average values of z-scored front-back (x, bottom row) and high-low (y, top row) positions of the highest point of the tongue in V**_**1**_**, across V**_**2**_ **contexts (/a/ or /i/), for both participant groups (red columns: Adults, blue columns: Children).** Left-hand panels: tokens for which V_1_ = /a/; right-hand panels: tokens for which V_1_ = /ε/. Error bars are standard errors of the mean.

Turning now to articulatory data, results of the first analysis of tongue position, based on the front-back and high-low positions of the highest point of the tongue contour, are depicted in Fig 9. It shows that adults displayed anticipatory patterns of V_2_ in V_1_ in both spatial dimensions, which was particularly clear when V_1_ = /a/, while children did not display this anticipation, and there was even a trend in the opposite direction for the children’s front-back dimension when V_1_ = /ɛ/. Linear mixed effects models conducted separately in the high-low and front-back dimensions confirmed the existence of a significant interaction between the fixed effects speaker group, V_1_ and V_2_ (high-low: χ^2^(4) = 53.28; p<0.001—front-back: χ^2^(4) = 16.55; p<0.01).

To further explore this interaction, multiple comparisons of V_2_ for tongue position, groups, and V_1_ values were conducted and the results are summarized in Table 2. For adults, there was a significant anticipatory effect of V_2_ on tongue height (1^st^ and 2^nd^ rows), both when V_1_ = /a/ and V_1_ = / ε /, with V_2_ = /i/ inducing higher positions than V_2_ = /a/, but there was a significant anticipatory effect on tongue frontness only when V_1_ = /a/, with a more anterior position for V_2_ = /i/ (3^rd^ and 4^th^ rows). For children, no effect of anticipation of V_2_ in V_1_ was found, either in tongue height or frontness (5^th^ to 8^th^ rows).

**Table 2:** Results of comparisons of V_2_ for the coordinates of the highest point of the tongue, according to the speaker group (adults and children) and the V_1_ level (V_1_ = /a/ and V_1_ = / ε).

Comparison	Estimate	Standard error	z value	Significance
**Adults high-low (y) in V1**				
/aCi/ vs. /aCa/	1.231	0.102	12.083	***
/εCi/ vs. /εCa/	0.306	0.098	3.135	**
**Adults front-back (x) in V1**				
/aCi/ vs. /aCa/	0.559	0.167	3.345	**
/εCi/ vs. /εCa/	0.013	0.161	0.082	ns
**Children high-low (y) in V1**				
/aCi/ vs. /aCa/	0.222	0.093	2.401	ns
/εCi/ vs. /εCa/	0.117	0.097	1.208	ns
**Children front-back (x) in V1**				
/aCi/ vs. /aCa/	0.220	0.110	2.001	ns
/εCi/ vs. /εCa/	-0.168	0.115	-1.458	ns

Significance abbreviations: ns = nonsignificant; * = p<0.05

** = p<0.01

*** = p<0.001

Importantly, the observed variations of the coordinates of the highest point of the tongue (Fig 9 and Table 2) were not consistent with the acoustic variations presented in Fig 7 and Table 1. This may have been due to the limited description of tongue shape based on a single point.

#### 4. Entire tongue contours

**Fig 10:**
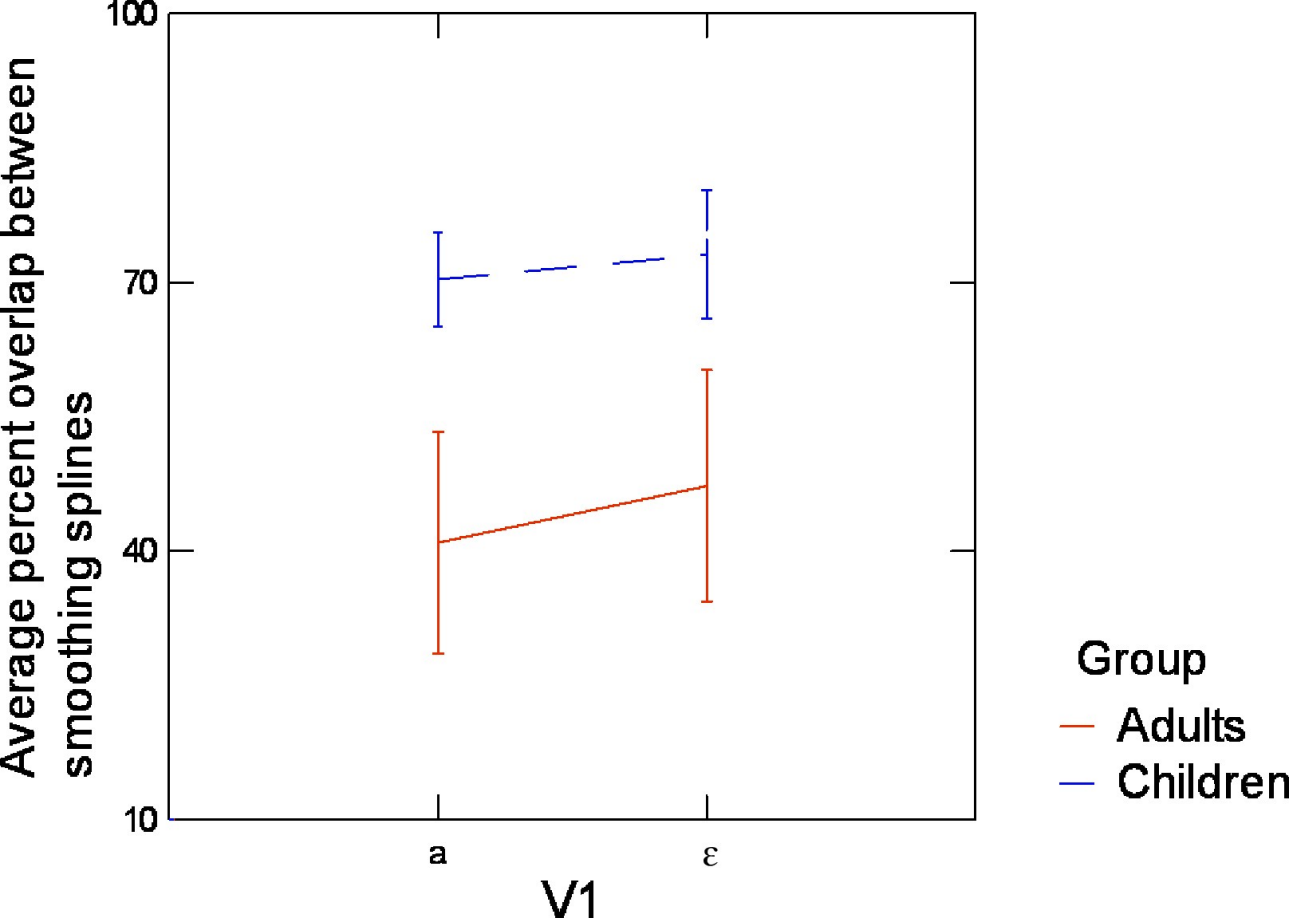
Average overlap across consonantal contexts between the confidence intervals of the average smoothing splines for V_1_ in both V_2_ contexts, for children and adults. Error bars are standard errors of the mean.

To better characterize tongue shapes, entire tongue contours, extracted from ultrasound data, were represented by average smoothing splines plus confidence intervals. To measure the variability in V_1_ associated with variation in V_2_ across repetitions and contexts, we quantified the overlap between confidence intervals of the average smoothing splines in V_1_ when V_2_ = /a/ and when V_2_ = /i/. The percentage of points, along the x axis, for which overlap occurred was taken as a measure of the overlap. The larger the overlap value, the smaller the variation associated with V_2_ and then the magnitude of anticipatory coarticulation. This measure is displayed in Fig 10.

Fig 10 shows that for both V_1_ vowels children present a larger amount of overlap between the confidence intervals of the average tongue contours of V_1_ respectively measured for V_2_ = /i/ and V_2_ = /a/ than adults (+31.07 for V_1_ = /a/ and +27.94 for V_1_ = /ɛ/). The large value of the overlap (around 70%) provides evidence that children did not differentiate much V_1_ articulation depending on V_2_. The differentiation was clearly stronger in adults, and this shows that adults had significantly greater anticipation than children. A linear mixed-effects model conducted with speaker group and V_1_ as fixed effects and subjects as random effects confirmed a significant effect of speaker group on the percent overlap (χ^2^(1) = 3.962; p<0.05) with no significant difference between V_1_ = /ɛ/ and V_1_ = /a/, indicating again that adults had significantly greater anticipation than children.

**Fig 11:**
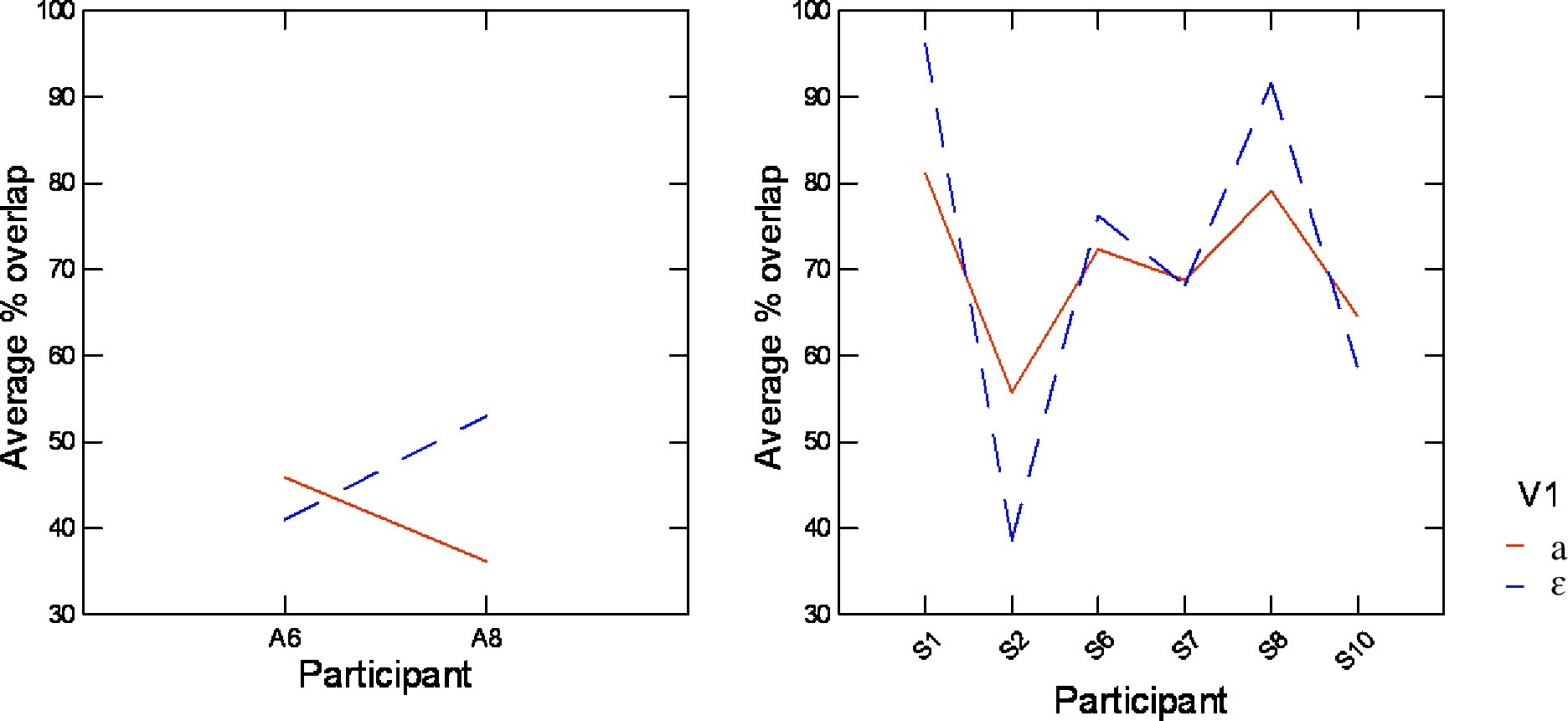
Average percent overlap in V_1_ between V_1_C/i/ and V_1_C/a,/ per participant. The solid line corresponds to /a/CV_2_ sequences and the dashed line corresponds to /ɛ/CV_2_ sequences.

As we did for the acoustic values, we analyzed each participant's behavior (2 adults and 6 children). Those data are displayed in Fig 11, for adults (left-hand panel) and children (right-hand panel). Importantly, the variability exhibited by children mirrored that found at the acoustic level (Fig 8). Indeed, in Fig 11, speaker C1 showed the largest amount of overlap, a pattern suggestive of reduced anticipatory coarticulation, as was seen in Fig 8. Speaker C2 had the smallest amount of overlap, in the range of the overlap found in adults, which is consistent with the observation in the acoustic domain that this child has adult-like anticipation (Fig 8). The same observation can be made for speakers C6 and C8. Speakers S7 and S10 had intermediate values of percent overlap, close to (but still above) those seen in adults, and this is also consistent with the observations in the acoustic domain.

In sum our articulatory measure of coarticulation based on the overlap between confidence intervals around the average spline approximations of the tongue contours for vowels V_1_ vs. V_2_ agrees with the measure of coarticulation that we provided in the acoustic domain. This consistency across the measures in different domains strengthens our conclusion that anticipatory coarticulation across the syllable boundary in V_1_CV_2_ sequences tends not to exist in children, or, in the rare cases where it does exist, to be clearly smaller than in adults.

## IV. Discussion

This study of speech production in 4-year-old children and in adults was designed to quantitatively assess the hypothesis that motor control immaturity might explain many of the observed differences between speech production in children and adults. We recorded acoustic and articulatory data and analyzed variables that are generally used to describe immaturity of motor control: (1) trial-to-trial variability across repetitions of the same motor task, (2) expression of anticipatory behavior in terms of anticipatory coarticulation, and (3) the duration of the movement and its variability. Based on prior observations in children for non-speech motor tasks, we predicted that children would display larger trial-to-trial variability in acoustic, articulatory and durational measures of speech production than adults, with longer movement durations and reduced anticipatory behavior compared to adults.

The articulatory point data and tongue contour data from a subset of 6 children and 2 adults were very consistent with the acoustic data (the F1 and F2 formants) from the cohorts of 20 children and 10 adults. Taken together, the data from this study clearly support the hypothesis that speech motor control immaturity plays a major role in the characteristics of speech production in 4-year-old children.

More specifically, we found that: (1) acoustic and articulatory variability measured in isolated vowel productions were on average around 1.5 times larger in children than in adults, which was statistically significant; (2) adults produced systematic anticipation of V_2_ in V_1_, independent of the identity of V_1_, along both the antero-posterior direction (associated with F2) and the open/close direction (associated with F1), whereas, with one exception, there was no significant anticipation in children. (The one exception to this observation for children was in the acoustic domain along the F2 dimension when V1 = /a/, and in this case the magnitude of the anticipation was much smaller than in adults); (3) durations of V_1_CV_2_ sequences were on average around 1.5 times longer in children than adults, which was also statistically significant.

The trial-to-trial variability in vowel production and movement duration of V_1_CV_2_ confirmed findings of prior acoustic and/or articulatory studies of speech production in children younger than 6. However, we believe that our characterization of anticipation is new and important. We interpret the reduced anticipatory coarticulation in 4-year-old children as evidence that these children did not take advantage, to the same extent as adults, of the degrees of freedom that characterize the relation between motor commands and auditory or somatosensory feedback: children either did not or barely anticipated V_2_ in V_1_, which suggests that they did not tend to minimize their articulatory effort by reducing the amount of articulatory displacement required for the production of the V_1_CV_2_ sequence. This is an important finding, since the large trial-to-trial variability observed in children in the production of isolated vowels may indicate that sensory goals associated with phonemes in children are larger than in adults. Thus the constraints in the production of the phonemes seem to be looser in children than in adults, and we could expect the children to use this larger tolerance for variability when adapting to different contexts. In agreement with our hypotheses based on the “parallel distributed processing” concept of speech motor planning [50], all these observations indicate that 4-year-old children either do not yet have the requisite skill for processing the sequence of speech motor goals in parallel along different articulatory dimensions, or that they cannot rely on sensorimotor maps that are sophisticated enough to enable an adult-like parallel processing, possibly because the maps are not yet comprehensive enough to account for all the degrees of freedom of the speech motor system.

The analysis of subject-specific results in anticipation of V_2_ along the antero-posterior direction sheds interesting light on this issue. This analysis showed that among adults, there were only small differences in vowel anticipation along the antero-posterior direction, but the behaviors of the children were very heterogeneous, in that a few children showed adult-like vowel anticipation while most others did not. Consistent with studies of non-speech motor control (for example [74] this observation suggests that age 4 may be at the beginning of a key period in speech motor control development that extends into and through adolescence. In our cohort of 4-year-old children, some precocious children seemed to have started the process that would lead to vowel anticipation seen in adults.

The combined analysis of the acoustic data for V_1_ = /a/ and V_1_ = / ε / showed that for most children, there was a trend towards anticipating V_2_ along the F2 dimension, although this did not reach statistical significance. This suggests that 4-year-old children may rely on some capacity to achieve a parallel processing in planning successive motor goals in sequence. This could arise from a better characterization of the sensorimotor relations in their sensorimotor maps, which could be the consequence of the greater frequency of vowel /a/ compared to /ε/, which in turn could have induced a larger sensorimotor experience around /a/ than around /ε/.

An alternative explanation for the general lack of anticipatory behavior in vowel production in 4-year-old children may be the longer duration of their articulatory movements. Indeed, there may be less need for anticipation when there is a long time to execute articulatory movements, whereas anticipation may be necessary to correctly achieve articulatory movement when time is short. We cannot discount this explanation, but we do not believe that it applies here, because we did not observe any significant correlation between the Euclidian distance separating the centers of the dispersion ellipses of V1 and V2 in the z-scored (F1,F2) plane (smaller distances would be evidence for more anticipation) and duration of the speech sequence, in either cohort (Adults, R^2^ = 0.6016, p<0.06576; Children, R^2^ = 0.207, p<0.3812). Therefore, speech was likely produced at a comfortable rate, which allowed the subjects to anticipate or to not anticipate the next vowel without endangering the acoustic-phonetic integrity of their utterances. Instead, we suggest that the longer durations observed in children may be an additional consequence of their undeveloped motor skills or the inefficiency of their sensorimotor maps. In adults, skilled motor control has been suggested to rely heavily on feedforward or feedback control based on quasi-instantaneous internal predictions of sensory outputs based on motor commands [75]. In the absence of the capacity to accurately provide these predictions, children would not be able to rely on predictive motor control and would have to rely on longer feedback loops, which induce slower movements and longer durations. Taken together, these observations are all consistent with the hypothesis that the speech production characteristics of 4-year-old children are strongly impacted by the incompleteness or the inefficiency of their sensorimotor maps, which prevents them from exploiting accurate predictions of the impact of motor commands on the articulatory and acoustic characteristics of their speech production.

Our study also showed that if we do not take into account the entire contour of the tongue, as we did using spline approximations, there may be some incongruence between anticipatory effects observed in the acoustic domain and those observed in the articulatory. This is what we observed when we used only the position of the highest point of the tongue to quantify coarticulation in the articulatory domain, as done in former studies of coarticulation in the literature. Specifically, this measure did not reveal any significant anticipatory effect in articulatory data in adults along the front/back dimension in V_1_ = /ε/, whereas anticipation was clearly observed along the F2 dimension in the acoustic domain. A potential explanation for this is the imprecision of measuring and using the horizontal position of the highest point of the tongue, when the tongue is not clearly bunched, as it is for vowels / ε / and /a/. Indeed, for a flat tongue, there is unavoidably a large variance across repetitions in the determination of the highest point of the contour, which in turn makes statistical significance more difficult to demonstrate.

To our knowledge, this is the first study in which both formant values and articulatory data were used to assess extra-syllabic anticipatory patterns in children. An important strength of the present study is the consistency of the results in the acoustic and articulatory domains, especially given the inconsistency in the literature about this topic. Some studies suggest that there is more vowel anticipation in children than in adults, whereas others (sometimes from the same authors) suggest that there is less. Our combined acoustic and articulatory findings provide strong support for the hypothesis that children show less vowel anticipatory coarticulation than adults.

## V. Conclusion

The present acoustic and articulatory study of trial-to-trial variability and anticipatory coarticulation supports the hypothesis that differences between speech production in adults and 4-year-old children are due in a large part to immaturity of motor control, which, in turn is characterized by incomplete or underspecified sensorimotor maps that link motor commands with sensory feedback. The variability across the child participants suggests that age 4 is at the onset of a key period that extends into adolescence and beyond, during which children develop sensorimotor maps and learn how to use them as they evolve toward using adult-like speech motor control strategies. We do not discount the potential role of phonological awareness (and of the role of the syllable in early children's speech in this process), which also starts around age 4–5 when children go to preschool and begin the rudiments of reading. Future work is needed to assess this potential link between motor skills and speech production by looking at phonological development as one of the factors that influence speech motor control skills.
